# The positive feedback between Snail and DAB2IP regulates EMT, invasion and metastasis in colorectal cancer

**DOI:** 10.18632/oncotarget.4861

**Published:** 2015-08-21

**Authors:** Jianmei Wang, Xiaohui Zhu, Jinlong Hu, Guoyang He, Xiaomei Li, Pingxiang Wu, Xiaoli Ren, Feifei Wang, Wenting Liao, Li Liang, Yanqing Ding

**Affiliations:** ^1^ Department of Pathology, Nanfang Hospital, Southern Medical University, Guangzhou 510515, Guangdong Province, People’s Republic of China; ^2^ Guangdong Province Key Laboratory of Molecular Tumor Pathology, Guangzhou 510515, Guangdong Province, People’s Republic of China; ^3^ Department of Pathology, The Affiliated Hospital of Luzhou Medical College, Luzhou 646000, Luzhou, China

**Keywords:** DAB2IP, Ezh2, Snail, colorectal carcinoma, metastasis

## Abstract

DAB2IP has been identified as a tumor suppressor in several cancers but its oncogenic role and transcriptionally regulatory mechanisms in the progression of colorectal carcinoma (CRC) remain unknown. In this study, DAB2IP was down-regulated in CRC tissues and a valuable prognostic marker for survival of CRC patients, especially in the late stage. Moreover, DAB2IP was sufficient to suppress proliferation, epithelial-mesenchymal transition (EMT), invasion and metastasis in CRC. Mechanically, the linear complex of EZH2/HDAC1/Snail contributed to DAB2IP silencing in CRC cells. The study further proved that the positive feedback loop between Snail and DAB2IP existed in CRC cells and DAB2IP was required for Snail-induced aggressive cell behaviors. Finally, DAB2IP correlated negatively with Snail and EZH2 expressions in CRC tissues. Our findings reveal the suppressive role and a novel regulatory mechanism of DAB2IP expression in the progression of CRC. DAB2IP may be a potential, novel therapeutic and prognostic target for clinical CRC patients.

## INTRODUCTION

Colorectal carcinoma (CRC) is a major cause of cancer-related morbidity and mortality. In the Western countries, it is the second leading cause of death from cancer [1]. The incidence of colorectal cancer has increased in China recently [2]. Therefore, some new molecular markers are necessary to raise the efficiency of tumor diagnosis and to predict prognosis of the patients or even for therapeutic application.

DAB2IP, also known as ASK1-interacting protein-1 (AIP1) is a recently identified, novel member of the RasGAP family. DAB2IP directly interacts with the N-terminal domain of DAB2 protein, then form a unique protein complex and have a negative regulatory activity to the Ras-mediated signal pathway [3]. DAB2IP via its Ras-GAP activity, inhibits Ras-mediated cell survival signaling, causing cell growth inhibition [4]. On the other hand, DAB2IP functions as a positive regulator in cell apoptosis by mediating activation of the apoptotic kinase ASK1 [4, 5]. Consistent with its role as an inhibitor of cell survival and growth, DAB2IP expression is often down-regulated in several human cancers [6–9], which is frequently associated with the promoter hypermethylation [8–13] or enhancer of zeste homolog 2 (EZH2)-mediated transcriptional silencing [14, 15].

Recently, DAB2IP has been regarded as a critical suppressor in cancer progression. Down-regulation of DAB2IP results in cell invasion in bladder cancer and hepatocellular cancer [6, 7]. DAB2IP also inhibits epithelial mesenchymal transition (EMT) and metastasis in prostate cancer [16, 17]. Moreover, DAB2IP suppresses cancer stem cell properties via CD117-meidiated ZEB1 signaling pathway [18]. In our previous study, DAB2IP was screened as a potential metastasis suppressor by cDNA microarray [19, 20]. We further raised that low expression of DAB2IP contributed to malignant development and poor prognosis in hepatocellular carcinoma [7]. However, the potential oncogenic role and transcriptional regulatory mechanisms of DAB2IP in the progression of CRC has not been clearly elucidated.

In this study, we unveil the suppressive role of DAB2IP in the progression of CRC, in which we identify the novel mechanism of the positive feedback between Snail and DAB2IP regulating EMT, invasion and metastasis in CRC cells.

## RESULTS

### DAB2IP is sufficient to inhibit the proliferation, invasion and EMT of CRC cells

**Figure 1:**
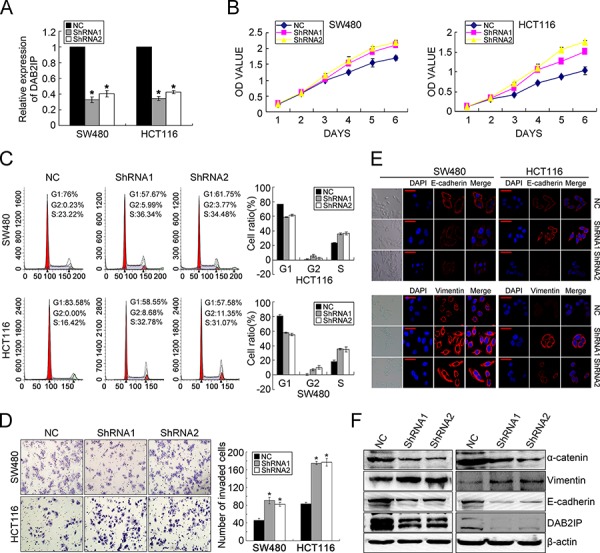
DAB2IP KD promotes proliferation, invasion and EMT in CRC cells **A.** DAB2IP expression in SW480 and HCT116 cell lines transfected with two specific shRNAs toward DAB2IP by real-time PCR. **B.** Effect of DAB2IP KD on the proliferation of SW480 and HCT116 cells by MTT assay. **C.** Effect of DAB2IP KD on cell cycle of SW480 and HCT116 cells by Flow Cytometry. **D.** Effect of DAB2IP KD on the invasion of SW480 and HCT116 cells by Boyden chamber. Scale bars represent 50 μm. **E.** Immunofluorescence analyses of E-cadherin and Vimentin in DAB2IP KD cells. Representative morphologic transition was also shown. Scale bars represent 10 μm. **F.** The expressions of E-cadherin, α-catenin and Vimentin in DAB2IP KD SW480 and HCT116 cells by Western blotting.

To characterize the oncogenic functions of DAB2IP in the progression of CRC, we investigated the effect of DAB2IP knockdown (KD) on CRC cell behaviors. According to endogenous DAB2IP expression in 7 CRC cell lines (Supplementary Figure S1A, S1B), we introduced two specific siRNAs toward DAB2IP into SW480 and HCT116 cell lines and generated stable transfectants (Figure 1A, Supplementary Figure S1C). Since the coding domain of DAB2IP gene is as long as 3399bp and rich in GC contents, we failed to construct the expressing vector. Our results showed that DAB2IP KD promoted the proliferation of SW480 and HCT116 cells by MTT assay (Figure 1B). Similar results were observed in colony formation assay (Supplementary Figure S1D). DAB2IP KD caused a significantly increased S or G2/M phase cell population, and a decreased cell population in G0/G1 phase in SW480 and HCT116 cells (Figure 1C), indicating that DAB2IP could elicit G0/G1 phase cell cycle arrest. However, DAB2IP KD did not affect cell apoptosis (Supplementary Figure S1E). DAB2IP KD also significantly enhanced cell invasion through Matrigel (Figure 1D), and promoted the migration in SW480 and HCT116 cells (Supplementary Figure S1F). However, it did not affect cell adhesion (Supplementary Figure S2A). When endogenous DAB2IP expression in SW480 and HCT116 cell lines was depleted, EMT was clearly detected based on changes in cell morphology and biomarker expression. DAB2IP KD SW480 and HCT116 cells displayed a clear morphological transition from spindle-like fibroblastic to cobblestone-like cells (Figure 1E). Meanwhile, DAB2IP KD increased the mesenchymal marker Vimentin and concomitantly reduced epithelial markers E-cadherin and α-catenin (Figure 1E, 1F). Taken together, these data indicate that DAB2IP is sufficient to suppress the proliferation, invasiveness and EMT of CRC cells *in vitro*.

### DAB2IP inhibits tumor growth and metastasis of CRC cells

**Figure 2:**
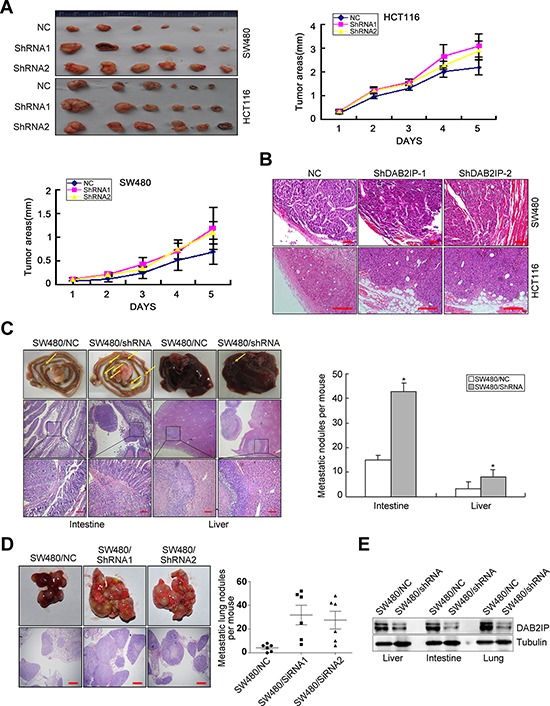
DAB2IP is sufficient to inhibit tumor growth and metastasis of CRC cells **A.** Effect of DAB2IP KD on subcutaneous tumor growth by MTT assay. **B.** Local invasion of subcutaneous tumors in DAB2IP KD group and control group. Small scale bars represent 100 μm. **C.** Images of intestinal and hepatic metastases of mice injected with DAB2IP KD SW480 cells (*n* = 5), and number of metastatic intestinal or hepatic nodules per mice. Small scale bars represent 200 μm. **D.** Image of pulmonary metastases of mice injected with DAB2IP KD SW480 cells, and number of metastatic lung nodules per mice. Scale bars represent 500 μm. **E.** Expression of DAB2IP in liver, intestinal or lung metastatic tumors in mice injected with DAB2IP KD cells and control cells by Western blotting. *P < 0.05.

Because of its effects on vitro traits associated with high-grade malignancy, we asked whether DAB2IP KD promotes metastasis in CRC cells. DAB2IP KD SW480 and HCT116 cells were subcutaneously injected into nude mice. Unexpectedly, DAB2IP KD enhanced subcutaneous tumor growth (Figure 2A) and correspondingly increased cell proliferation labeled by Ki67 index (Supplementary Figure S2B). Control primary tumors were well encapsulated and noninvasive, while DAB2IP KD tumors displayed evidence of local invasion (Figure 2B).

To test the effect of DAB2IP on CRC metastasis *in vivo*, we adopted orthotropic metastasis nude mouse model. Orthotopic transplant of DAB2IP KD SW480 cells into nude mice showed a significant increase in the number and size of spontaneous liver and intestinal metastases (Figure 2C). We also injected DAB2IP KD SW480 cells into the tail vein of nude mice to examine the effect of DAB2IP on lung colonization *in vivo*. DAB2IP KD SW480 cells strikingly enhanced their capacity to seed lung metastases (Figure 2D). The number of metastatic lesions in DAB2IP KD group was significantly more than control group (*P* < 0.05, Figure 2D). The expression of DAB2IP was obviously decreased in hepatic, intestinal or pulmonary metastatic tumors in mice injected with DAB2IP KD cells (Figure 2E). These data make it obvious that DAB2IP inhibits tumor growth and metastasis of CRC cells *in vivo*.

### Snail negatively regulates DAB2IP expression

**Figure 3:**
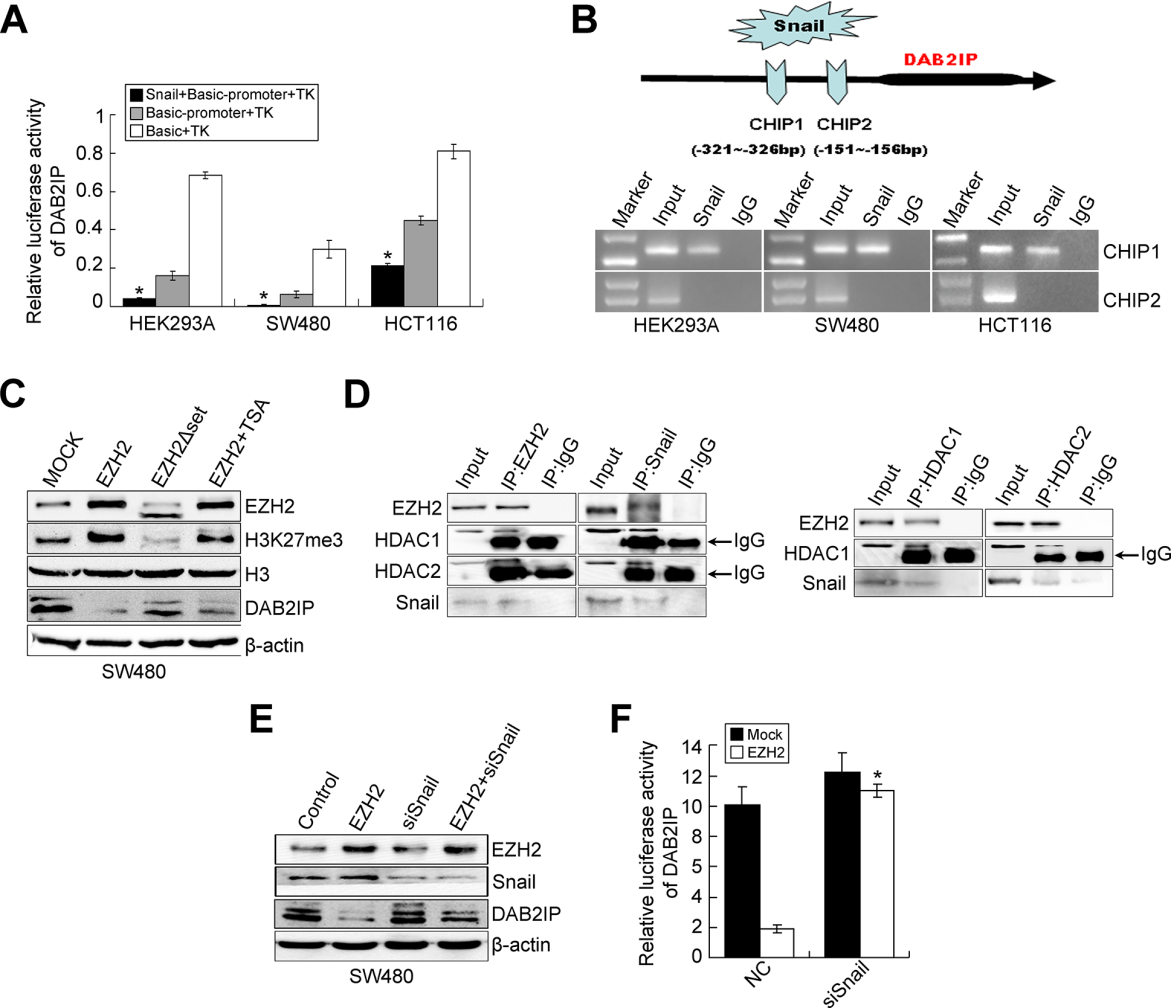
Snail negatively regulates DAB2IP expression **A.** Luciferase activity of DAB2IP promoter after transfection of Snail plasmid in HEK293A, SW480 and HCT116 cells. **B.** ChIP assay in HEK293A, SW480 and HCT116 cells transfected with a vector expressing Snail. PCR was performed with primers specific for two binding sites (CHIP1 and CHIP2) within DAB2IP promoter. **C.** Effects of ectopic EZH2, EHZ2ΔSET and EZH2 with TSA treatment on the expressions of DAB2IP, H3 and H3K27me3 in SW480 cells by Western blotting. **D.** The physical interactions between HDAC1/2 and EZH2, HDAC1/2 and Snail in SW480 cells examined by co-IP. **E.** Expressions of EZH2, Snail and DAB2IP in EZH2-overexpressing, Snail-KD or EZH2-overexpressing/Snail-KD cells by Western blotting. **F.** Effect of Snail KD on luciferase activity of DAB2IP after transfection of EZH2 plasmid in SW480 cells.

Down-regulation of DAB2IP in several cancers is mainly due to altered epigenetic regulation of its promoter [10–17]. However, the transcriptional regulation of DAB2IP in cancer has not been illustrated. We first detected the expression of DAB2IP in 20 paired cases of fresh CRC tissues. DAB2IP was markedly down-regulated in CRC tissues compared to adjacent normal mucosa (*P* = 0.012, Supplementary Figure S2C). DAB2IP protein expression in CRC tissues coincided precisely with that of the mRNA level (Supplementary Figure S2D). Next, we analyzed the potential transcription factors binding the 1kb region (promoter) upstream of DAB2IP by Mapper2 (http://genome.ufl.edu/mapperdb), Consite (http://consite.genereg.net/) and TRED (http://rulai.cshl.edu/cgi-bin/TRED/tred.cgi?process=home) databases. All databases predicted presence of two possible binding motifs for snail within the promoter of DAB2IP (Supplementary Figure S2E). It was observed that transient expression of snail effectively inhibited the transcription activity of DAB2IP in SW480, HCT116 and HEK293A cells (*P* < 0.01, Figure 3A). Results of ChIP then validated that snail could bind the region of R1 in the promoter of DAB2IP (Figure 3B). Moreover, ectopic snail led to reduced expression of DAB2IP, while knockdown of snail increased DAB2IP expression (Supplementary Figure S2F). These results suggest that snail down-regulates the transcriptional level of DAB2IP.

### The complex of EZH2/HDAC/Snail contributes to DAB2IP silencing in CRC cells

Recent studies provide evidence that down-regulation of DAB2IP is mediated by polycomb EZH2 and histone deacetylase in prostate cancer [16, 17]. EZH2 negatively regulates E-cadherin expression via trimethylation of H3K27 [23, 24] and transcriptional factor snail is required for EZH2-mediated E-cadherin repression in nasopharygeal cancer cells (NPC) [24]. So we speculated that EZH2 may interact with HDAC1/HDAC2 and snail to repress DAB2IP in CRC cells. We first examined whether EZH2 regulates DAB2IP expression in CRC cells. Since the SET domain of EZH2 is required for EZH2-mediated E-cadherin regulation [23, 24], we generated the SET domain deletion mutation of EZH2 (EZH2ΔSET) (Supplementary Figure S3A). The results showed that ectopic EZH2 caused a reduced expression of DAB2IP in SW480 cells, accompanied by increased trimethylation of H3K27me3 (Figure 3C), while the introduction of EZH2ΔSET did not have the same effect (Figure 3C). The treatment of Trichostatin A (TSA), a HDAC inhibitor, partially restored the reduced level of DAB2IP in EZH2-expressing cells (Figure 3C). However, EZH2 KD increased the level of DAB2IP in SW480 and HCT116 cells (Supplementary Figure S3B). These results demonstrate that EZH2 negatively regulates DAB2IP expression in CRC cells and the EZH2-mediated DAB2IP repression requires HDAC activity. Next, we performed a co-IP assay to test whether EZH2, HDACs and snail can interact virtually in CRC cells. The existence of snail, HDAC1 or HDAC2 was detected in the immunoprecipitates obtained with antibody against EZH2. Similarly, we detected EZH2, HDAC1 and HDAC2 in snail immunoprecipitates (Figure 3D). Consistently, EZH2 and snail were present in HDAC1 or HDAC2 immunoprecipitates (Figure 3D). These results indicate that EZH2 interacts with HDAC1/HDAC2 and snail to form a multi-molecular complex. Subsequently, we investigated whether snail is required for EZH2-mediated DAB2IP silencing. The repression of EZH2 toward DAB2IP expression or transcriptional activity was virtually prevented in snail-depleting cells (Figure 3E, 3F). These results suggest that the presence of snail is required for the repressive function of EZH2 toward DAB2IP. Finally, we tested whether HDAC1/HDAC2 is necessary for the repressive function of EZH2 on the DAB2IP promoter. The repressive activity of ectopic EZH2 in the DAB2IP promoter was dramatically inhibited after the depletion of endogenous HDAC1 or HDAC2. The repressive activity with combined treatment with siHDAC1 and siHDAC2 was obviously enhanced compared with that of each alone (Supplementary Figure S3C, S3D). Collectively, the above data indicate that EZH2, HDAC1/HDAC2 and snail form a co-repressor complex to silence DAB2IP in CRC cells.

### DAB2IP is negatively regulated by EZH2/HDAC/snail linear transcription complex

**Figure 4:**
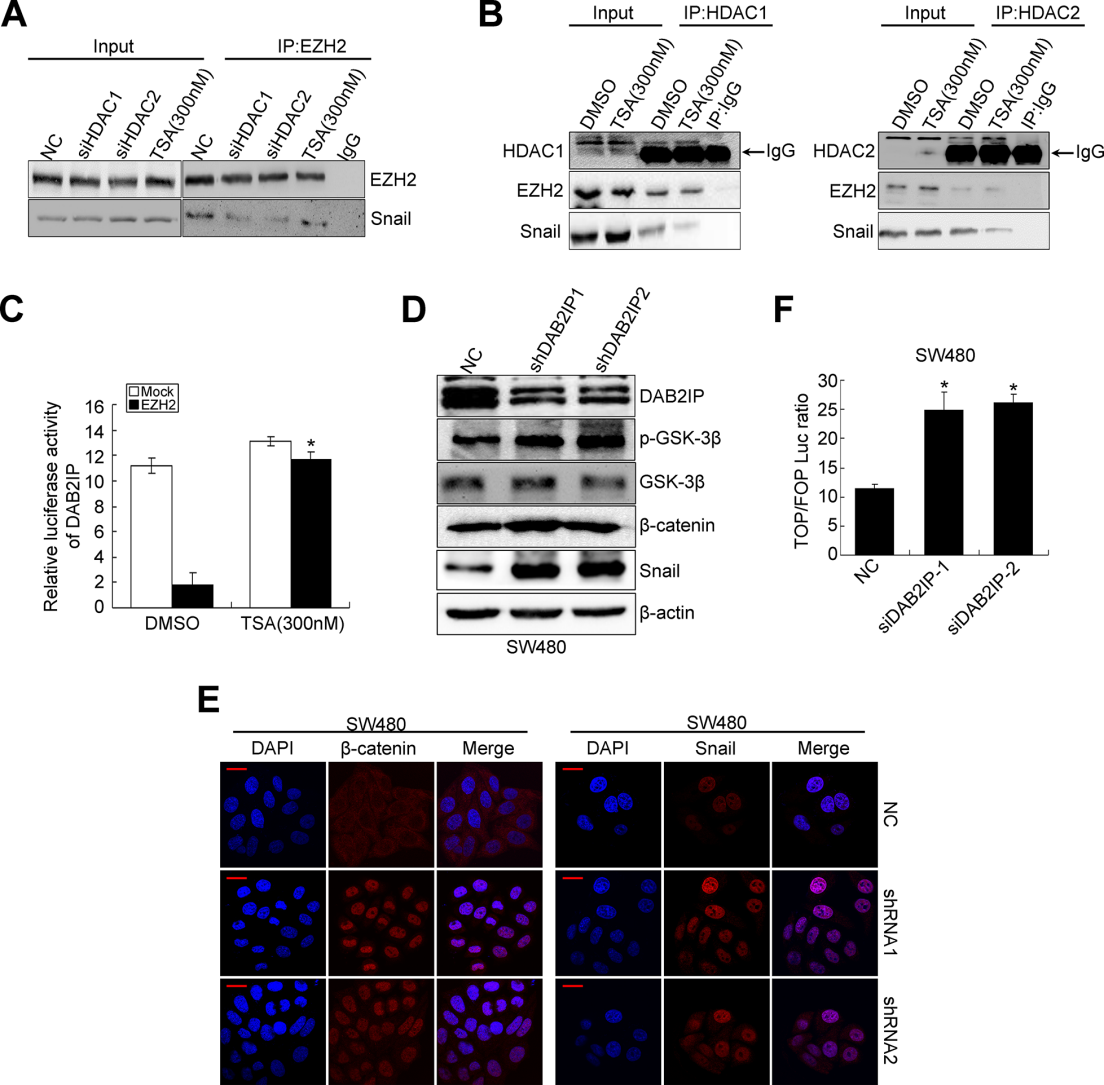
DAB2IP is negatively regulated by EZH2/HDAC/snail linear transcription complex **A.** Effects of HDAC1/2 knockdown, TSA treatment on the physical interactions between EZH2 and Snail by co-IP. **B.** Effects of TSA treatment on the physical interactions between HDAC1/2 and EZH2, HDAC1/2 and Snail by co-IP. **C.** Effect of TSA on the luciferase activity of DAB2IP promoter after transfection of EZH2 plasmid in SW480 cells. **D.** Expressions of DAB2IP, p-GSK-3β, GSK-3β, β-catenin and Snail in DAB2IP KD SW480 cells. **E.** Confocal microscopy visualization of subcellular localization of β-catenin and Snail in DAB2IP KD SW480 cell. Scale bars represent 10 μm. **F.** TOP/FOP luciferase activity after transfection of DAB2IP siRNAs in SW480 cells.

Since HDAC1/2 mediates EZH2 and snail interaction in NPC cells [24], we performed co-IP experiment to examine whether HDAC1/HDAC2 bridges the interaction between EZH2 and Snail in CRC cells. Either siHDAC1 or siHDAC2 inhibited the interaction between EZH2 and Snail (Figure 4A). We also treated CRC cells with TSA (HDAC inhibitor) and analyzed the amount of Snail in the EZH2 immunoprecipitates. As anticipated, TSA treatment inhibited the interaction between EZH2 and Snail (Figure 4A). These results suggest that the association of EZH2 and Snail is dependent on HDAC activity. Next, to determine which of the association of HDAC1/2 and EZH2 or HDAC1/2 and Snail is affected by TSA, we performed co-IP experiments and found that TSA significantly decreased the interaction between HDAC1/2 and Snail, but did not affect the association of HDAC1/2 and EZH2 (Figure 4B). Consistently, TSA blocked the repressive activity of ectopic EZH2 on the DAB2IP promoter in SW480 cells (Figure 4C). These observations, taken together, provide substantial evidence that EZH2, HDAC1/2 and Snail form a linear complex to decrease the expression of DAB2IP in CRC cells.

### The positive feedback loop between snail and DAB2IP exists in CRC cells

In prostate cancer, DAB2IP functions as a scaffold protein in regulating EMT through GSK3β-β-catenin signaling pathway [18]. In the process of EMT, DAB2IP KD failed to recruit PP2A to active GSK-3β through its C2 domain, leading to increased nuclear β-catenin transcriptional activity [18]. Snail is highly unstable and GSK-3β binds to and phosphorylates Snail at two consensus motifs to dually regulate the function of this protein. Inhibition of GSK-3β suppresses the phosphorylation of Snail and thus induces the protein stabilization and nuclear localization of Snail, which leads to EMT [25]. Therefore, we sought to examine whether DAB2IP regulates the stability and subcellular localization of Snail in SW480 cells. Indeed, DAB2IP KD induced high Ser9 (S9, negative regulatory site) phosphorylation level of GSK-3β (Figure 4D), nuclear translocation of β-catenin (Figure 4E) and transcriptional activation of TCF/LEF (Figure 4F). Moreover, it increased the protein level and nuclear translocation of Snail (Figure 4D, 4E). In addition, DAB2IP KD markedly repressed the transcription activity toward DAB2IP (Supplementary Figure S3E), which further validated that DAB2IP negatively regulated Snail. These findings suggest that DAB2IP is sufficient to reduce the expression and nuclear translocation of Snail.

**Figure 5:**
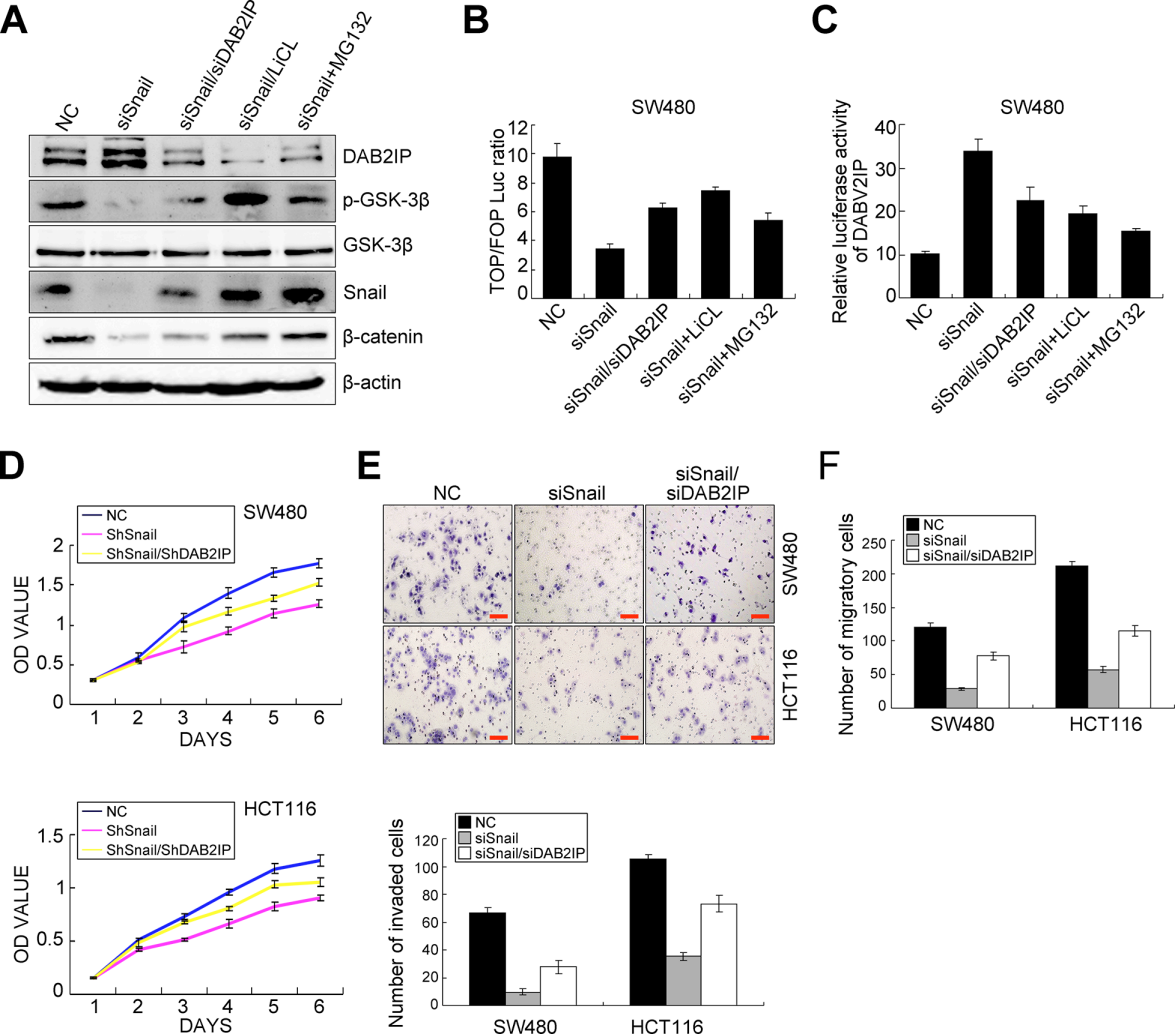
The positive feedback loop between Snail and DAB2IP exists in CRC cells **A.** Expressions of DAB2IP, p-GSK-3β, GSK-3β, Snail and β-catenin in Snail KD SW480 cells and Snail/DAB2IP KD SW480 cells with LiCL, MG132 treatments by Western blotting. **B.** TOP/FOP luciferase activity in Snail KD SW480 cells and Snail/DAB2IP KD SW480 cells with LiCL, MG132 treatments. **C.** Luciferase activity of DAB2IP promoter in Snail KD SW480 cells and Snail/DAB2IP KD SW480 cells with LiCL, MG132 treatments. **D.** Effects of Snail and Snail/DAB2IP KD on the proliferation of SW480 and HCT116 cells by MTT assay. **E.** Effect of Snail and Snail/DAB2IP KD on the invasiveness of SW480 and HCT116 cells by Boyden chamber. Scale bars represent 50 μm. **F.** Effect of Snail and Snail/DAB2IP KD on the migration of SW480 and HCT116 cells by Transwell chamber.

Since Snail was not only an upstream modulator, but also a downstream target of DAB2IP in CRC cells, we speculated that Snail and DAB2IP may constitute a positive feedback loop in CRC cells. To test this hypothesis, we knocked down Snail in SW480 and HCT116 cells and rescued the expression of DAB2IP KD in those cells (Supplementary Figure S3F). Snail KD reduced the phosphorylation level of GSK-3β (S9), protein content of β-catenin (Figure 5A), transcriptional activity of TCF/LEF (Figure 5B) and enhanced the transcription activity of DAB2IP in SW480 cells (Figure 5C). However, depletion of DAB2IP, at least partially, reversed these effects resulting from Snail KD in SW480 cells (Figure 5A–5C). To dissect whether the positive feedback between DAB2IP and Snail modulates Snail stability via proteasome-dependent ubiquitination mediated by GSK3β in SW480 cells, we treated Snail KD cells with LiCL (inhibitor of GSK-3β) or MG132 (a proteasome inhibitor). Unexpectedly, LiCL treatment obviously increased the phosphorylation level of GSK-3β and the expressions of β-catenin, Snail (Figure 5A) and transcriptional activity of TCF/LEF in Snail KD cells (Figure 5B). However, it reduced the transcription activity of DAB2IP in Snail KD cells (Figure 5C). The similar changes were also observed in Snail KD cells treated with MG132 (Figure 5A–5C). Since the stability of Snail is dependent on its interaction with β-Trap [26], we introduced β-Trap into Snail-expressing SW480 cells. Snail increased the phosphorylation level of GSK-3β, the protein level of β-catenin but decreased DAB2IP expression in SW480 cells, while reintroduction of β-Trap in Snail-expressing SW480 cells accelerated the degradation of Snail and subsequently induced up-regulation of DAB2IP, activation of GSK-3β and down-regulation of β-catenin (Supplementary Figure S4A). These results indicate that the positive feedback between Snail and DAB2IP mediates Trcp-mediated ubiquitination of Snail by inhibiting GSK-3β signaling in CRC cells. Thus, Snail and DAB2IP constitute a positive feedback loop in CRC cells.

### DAB2IP is required for Snail-induced proliferation, invasion, EMT and metastasis

**Figure 6:**
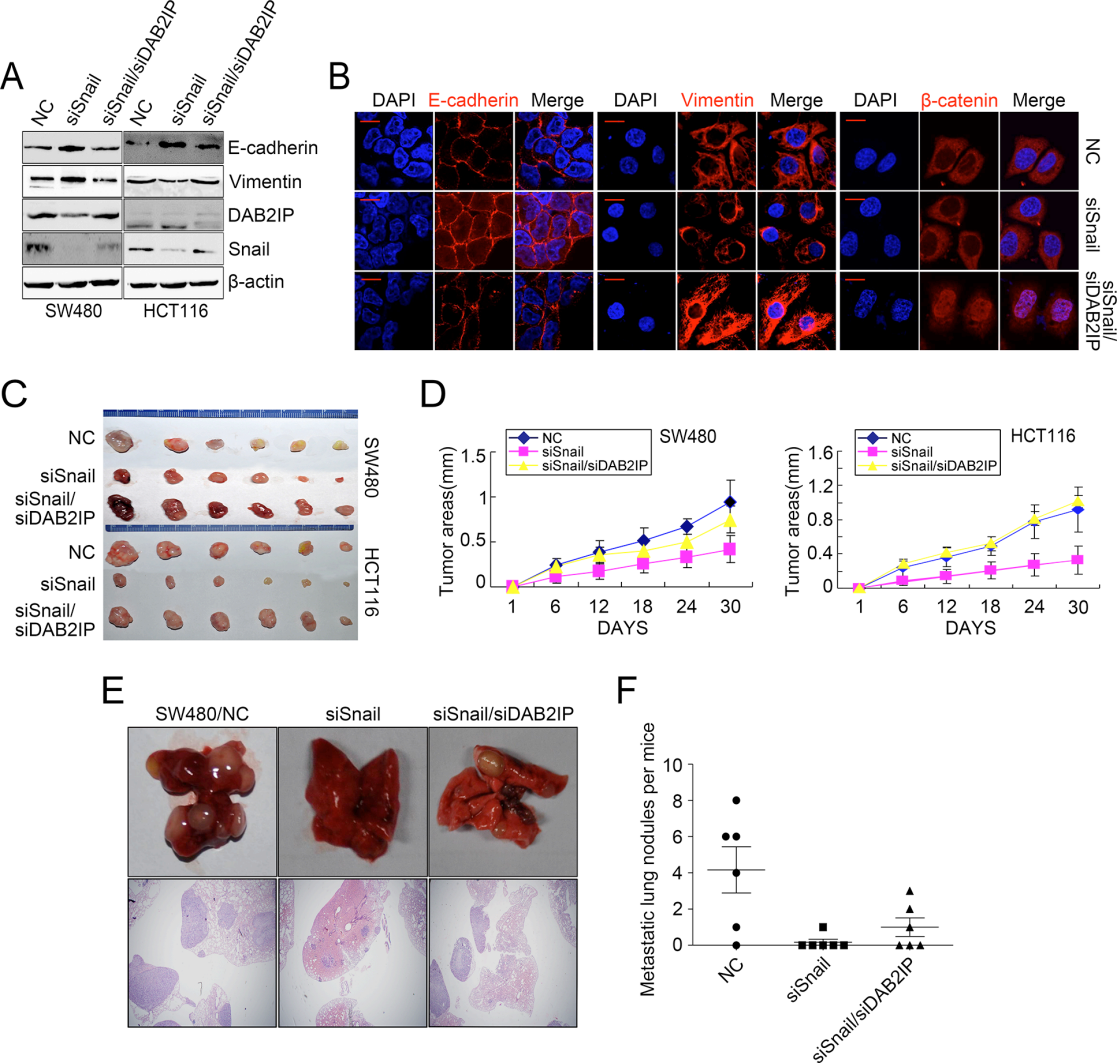
DAB2IP is required for Snail-induced proliferation, invasion, EMT and metastasis **A.** Expressions of E-cadherin, Vimentin, DAB2IP and Snail and Snail/DAB2IP KD cells by Western blotting. **B.** Immunofluorescence analyses of E-cadherin, Vimentin and β-catenin in Snail and Snail/DAB2IP KD cells. Scale bars represent 5 μm. **C.** Subcutaneous tumors of mice injected with Snail and Snail/DAB2IP KD SW480 and HCT116 cells. **D.** Effects of Snail and Snail/DAB2IP KD on subcutaneous tumor growth by MTT assay. **E.** Pulmonary metastases of mice injected with Snail and Snail/DAB2IP KD SW480 cells. Scale bars represent 500 μm. **F.** Effects of Snail and Snail/DAB2IP KD on pulmonary metastases.

To validate that snail mediated CRC progression was through DAB2IP, we examined the effect of blocking DAB2IP on Snail KD-induced proliferation, invasion, EMT and metastasis. As expected, Snail KD attenuated cell proliferation *in vitro* in SW480 and HCT116 cells, as shown by MTT and colony formation assays, while these effects were obviously abolished upon depletion of DAB2IP (Figure 5D, Supplementary Figure S4B). Snail KD also reduced the number of migratory and invaded CRC cells *in vitro*. However, reintroduction of DAB2IP KD alleviated the suppressive effects of Snail KD on SW480 and HCT116 cells (Figure 5E–5F). Snail KD suppressed EMT in SW480 and HCT116 cells, as shown by up-regulation of E-Cadherin and down-regulation of Vimentin (Figure 6A, 6B). It also reduced the nuclear translocation of β-catenin (Figure 6B). However, ectopic expression of DAB2IP KD rescued the Snail-deficient MET phenotypes (Figure 6A, 6B). In the mouse models of tumor growth and metastasis, Snail KD cells and Snail/DAB2IP KD cells were injected in subcutaneous site of nude mice or into tail vein to seed lung metastases, respectively. Snail KD SW480 and HCT116 cells inhibited the growth of subcutaneous tumors (Figure 6C–6D). Moreover, Snail KD SW480 cells strikingly suppressed their capacity to seed lung metastases (Figure 6E, 6F). However, reintroduction of DAB2IP KD reversed Snail KD-induced growth and metastasis (Figure 6C–6F). Based on these results it would be reasonable to conclude that Snail’s function on the proliferation, invasion, EMT and metastasis in CRC cells requires the presence of DAB2IP.

### Correlations of DAB2IP, Snail and EZH2 in CRC tissues

**Figure 7:**
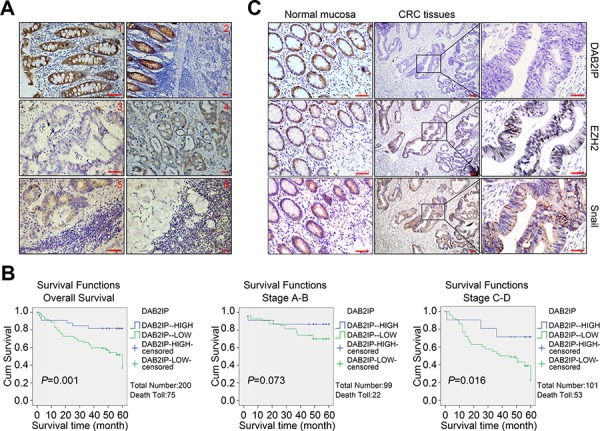
Down-regulation of DAB2IP correlates with Snail and EZH2 in CRC tissues **A1.** Strong positive staining of DAB2IP in the cytoplasm of adjacent normal mucosa. Scale bars represent 20 μm. **A2.** Down-regulation of DAB2IP in sections containing both normal and cancerous tissue. Scale bars represent 50 μm. **A3.** Negative staining of DAB2IP in the cytoplasm of CRC cells. Scale bars represent 20 μm. **A4.** Weak expression of DAB2IP in the cytoplasm of CRC cells. Scale bars represent 50 μm. **A5.** Weak expression of DAB2IP in lymphatic metastatic tissues of CRC. Scale bars represent 20 μm. **A6.** Negative expression of DAB2IP in lymphatic metastatic tissues of CRC. Scale bars represent 50 μm. **B.** Kaplan-Meier analysis of overall survival in total and subgroups of CRC patients. **C.** Expressions of DAB2IP, EZH2 and Snail proteins in normal mucosa and CRC tissues from the same specimens. Small scale bars represent 200 μm.

To investigate the expression pattern of DAB2IP and its clinical significance, we detected DAB2IP expression in 200 cases of CRC tissues by IHC. The staining signal of DAB2IP was observed mainly in the cytoplasm of adjacent normal mucosa epithelia (Figure 7A1, 7A2), and no signals or only weak signals were detected in CRC tissues and lymphatic metastatic tissues (Figure 7A3–7A6). Expression of DAB2IP was significantly lower in CRC tissues or lymphatic metastatic tissues than adjacent normal mucosa respectively (*P* < 0.01). DAB2IP was obviously down-regulated in CRC tissues with lymphatic metastasis compared to those without lymphatic metastasis (*P* < 0.01). DAB2IP expression was correlated strongly with differentiation, lymphatic metastasis, Duke’s stage and remote metastasis (*P* < 0.05, Supplementary Table S1). Notably, low DAB2IP protein level was a significant prognostic factor for poor overall survival of CRC patients (*P* = 0.001), especially in the subgroups of patients at the late clinical stage (stage III/IV, *n* = 101, *P* = 0.016, Figure 7B). Multivariate analysis results showed that DAB2IP expression and Duke’s stage might play a role in predicting the overall survival in CRC patients (*P* < 0.05, Supplementary Table S2). These above results confirm the low-expression of DAB2IP in CRC tissues and indicate that DAB2IP is a predictive biomarker for survival of CRC patients, especially at the late stage.

We next assessed the expression correlations of DAB2IP, EZH2 and Snail in 100 cases of CRC tissues. The positive signal of Ezh2 or Snail was distributed in the nucleus or both nucleus and cytoplasm of cells, respectively (Figure 7C). Expression of Ezh2 or Snail was significantly higher in CRC tissues or lymphatic metastatic tissues than adjacent normal mucosa, respectively (*P* < 0.05). Ezh2 was up-regulated in CRC tissues with lymphatic metastasis compared to metastatic CRC tissues in lymph nodes (*P* < 0.05). Across 100 cases of CRC tissues tested, we observed significant inverse correlations between DAB2IP and Ezh2 protein levels (*P* = 0.04, *r* = −0.333), DAB2IP and Snail protein levels (*P* = 0.01, *r* = −0.226). The above results prove that the epigenetic and transcriptional suppressions of DAB2IP by Ezh2 and Snail may be a major mechanism of its inactivation in CRC tissues.

## DISCUSSION

In this study, we first identify that DAB2IP functions as a tumor suppressor in the progression of CRC. We performed loss-of-function assay to assess the effect of DAB2IP on CRC cell behaviors. We found that DAB2IP was sufficient to elicit G0/G1 phase cell cycle arrest, which might account for the inhibition of cell proliferation in CRC cells. Moreover, DAB2IP knockdown enhanced EMT and invasion in CRC cells. The vivo results showed that DAB2IP was necessary for tumor growth and metastasis in CRC. In prostate cancer, the reciprocal regulation between DAB2IP and Skp2 can impact on the growth of prostate cancer cells [27]. Down-regulation of DAB2IP results in cell proliferation and invasion in bladder cancer and hepatocellular cancer [8, 9]. Moreover, loss of DAB2IP expression in prostate epithelia leads to EMT and metastasis [18, 19]. Our data also demonstrate a tumor suppressive role of DAB2IP in CRC progression.

Second, we show that both the transcriptional and epigenetic regulations contribute to down-regulation of DAB2IP in CRC cells. Evidences show that down-regulation of DAB2IP in cancer is mediated by polycomb EZH2 and histone deacetylase [16, 17]. EZH2, a central member of polycomb repressive complexes, has been found to contribute to the maintenance of cell identity, cell cycle regulation and oncogenesis [28]. However, the precise mechanisms by which EZH2 regulates DAB2IP expression and the transcriptional regulation of DAB2IP expression in CRC have not been illustrated. Our results showed that Snail negatively regulated DAB2IP expression in CRC cells. Snail is known to promote EMT and elicits associated pathological characteristics such as invasion, metastasis and stemness in cancer cells [29, 30]. Recently, EZH2 contributes to cancer cell invasion and metastasis by down-regulation of E-cadherin through trimethylation of H3K27 [31]. And snail is necessary for EZH2-mediated E-cadherin repression in NPC cells [24]. Moreover, snail mediates E-cadherin repression by the recruitment of the HDAC1/2 complex and E-cadherin is suppressed by a snail/HDAC1/HDAC2 complex to regulate metastasis of pancreatic cancer [32]. These results, taken together, prompted us to ask (1) whether or not EZH2 acts as a co-repressor complex with HDAC1/2 and snail to repress DAB2IP in CRC cells, and (2) if so, in which fashion does these components interact each other. Our results showed that the presence of snail was required for the repressive function of EZH2 toward DAB2IP. Moreover, EZH2, HDAC1/2 and snail formed a linear co-repressor complex to silence DAB2IP in CRC cells.

Third, we demonstrate that the positive feedback loop between snail and DAB2IP promotes invasion and metastasis in CRC. Other groups previously reported that DAB2IP suppressed EMT by modulating GSK3β/β-catenin signaling pathway in prostate cancer [18]. Inhibition of GSK-3β suppressed the phosphorylation of Snail and thus induced the protein stabilization and nuclear localization of Snail, which led to EMT [25]. Our results also showed that DAB2IP was sufficient to regulate GSK3β/β-catenin pathway and reduce the expression and nuclear translocation of snail. Thus, we raised the hypothesis that snail and DAB2IP might constitute a positive feedback loop in CRC cells. Unexpectedly, in snail depleting cells, DAB2IP KD rescued snail silencing-induced the inactivation of GSK-3β/β-catenin signaling and transcription activity of DAB2IP. We also proved that the positive feedback between DAB2IP and Snail mediated Trcp-mediated ubiquitination of Snail by inhibiting GSK-3β signaling in CRC cells. The functional rescue assays showed that reintroduction of DAB2IP KD abolished the suppressive effects of Snail KD on proliferation, invasion, EMT and metastasis in CRC cells, indicating DAB2IP was necessary for snail-induce CRC cell behaviors.

Finally, we provide evidence that down-regulation of DAB2IP in CRC tissues is a valuable prognostic marker for CRC patients and correlates with the expressions of snail and EZH2. To date, a significant prevalence of DAB2IP lost has been found in several different tumor types, including prostate cancer, hepatocellular cancer, breast cancer and lung cancer [9–11, 16]. However, the expression pattern of DAB2IP and its significance in CRC tissues remain unclear. Our results showed that DAB2IP was down-regulated in 200 cases of CRC tissues and a significant prognostic factor for poor overall survival of CRC patients, especially at the late clinical stage. We also observed significant inverse correlations between DAB2IP and Ezh2, DAB2IP and Snail protein levels in the same 100 paired cases of CRC tissues. The above results clearly validate that epigenetic and transcriptional suppressions of DAB2IP by Ezh2 and Snail may be a major mechanism of its inactivation in CRC tissues.

To conclude, we show a novel regulatory mechanism of DAB2IP expression in CRC wherein EZH2, HDAC1/2 and snail formed a linear co-repressor complex to silence DAB2IP in CRC cells. Moreover, the positive feedback loop between snail and DAB2IP promotes invasion and metastasis in CRC. Down-regulation of DAB2IP was a significant prognostic factor for CRC patients. Understanding the precise role played by DAB2IP in CRC progression not only will increase our understanding of the biology of CRC, but also may provide a potential, novel therapeutic molecular target for clinical CRC patients.

## MATERIALS AND METHODS

### Construction of plasmids and transfection

Lentiviral constructs repressing DAB2IP and Snail (Genechem, Shanghai city, China) were packaged using the pPACKH1 lentivector Packaging Kit (System Biosciences) and were used to infect CRC cells to establish stable cells. DAB2IP-siRNA1 (sense 5′-GGU GAA GGA CUU CCU GACA dTdT-3′), DAB2IP-siRNA2 (sense 5′-GGG AUA GGC UAA GGA GUAA dTdT-3′). Snail-siRNA1 (sense 5′-AAC UGC AAA UAC UGC AACA dTdT-3′). In the rescue experiments, Snail-depleting cells were transfected with shRNAs towards DAB2IP. For HDAC1/2 silence, specific siRNA duplexes toward HDAC1/2 were designed. HDAC1-siRNA1 (sense 5′-AAG CAG ATG CAG AGA TTC AAC-3′); HDAC2-siRNA (sense 5′-AAG CAT CAG GAT TCT GTT ACG-3′).

### Proliferation, plate colony formation, cell adhesion, cell cycle, apoptosis, cell migration, cell invasion assays *in vitro*

The proliferation, plate colony formation, cell adhesion, cell cycle, apoptosis, migration and invasion of transfected CRC cells were determined as previously described [21, 22].

### Animal models

For *in vivo* tumor growth assay, xenograft tumors were generated by subcutaneous injection of 1 × 10^7^ cells. Tumors were measured with calipers to estimate volume from day 5 to day 28 after injection. For tail vein metastasis assay, a total of 1 × 10^6^ cells were injected into the tail veins of nude mice. After 45 days, mice were killed, lung tissues were dissected and subjected to histological examination. For orthotropic metastasis assay, nude mice were anesthetized and their cecum was exteriorized by laparotomy. The subcutaneous tumors were embedded into the mesentery at the tail end of cecum. The gut was reposited to the abdominal cavity and closed with surgical drapes. Six weeks later, the mice were killed and all organs were removed for biopsy. Metastatic tumors were quantified by counting metastatic lesions in each section. Images were taken by Olympus DP72 upright microscope and were outputted by DP2-BSW software.

### Co-immunoprecipitation (Co-IP) analyses

Cell extracts were incubated 2 h at 4°C with IgG and protein A+G Agarose to get rid of unspecific binding. EZH2, HDAC1, HDAC2 (Cell Signaling Technology, Inc, USA) or Snail (Abcam, Cambridge, UK) antibody was then added at 4°C overnight. The protein A/G-agarose was collected by centrifugation. Immunoprecipitated proteins were analyzed by SDS-PAGE (12%, Minigel) followed by transfer to membranes at 100 V for 1.5 h. Membranes were blocked overnight. EZH2, HDAC1, HDAC2 and Snail antibodies were diluted respectively and incubated with membranes at 4°C overnight. The secondary antibodies were then incubated for 1 h at room temperature. Protein bands were visualized using enhanced chemiluminescence (PerkinElmer Life Sciences).

### Statistical analysis

The expressions of DAB2IP, Snail and Ezh2 in CRC tissues, adjacent normal mucosa and metastatic CRC tissues in lymph nodes were analyzed by Mann-Whitney test. The correlation of DAB2IP to various clinicopathological parameters was evaluated with χ^2^test. Survival analyses were performed according to the Kaplan-Meier method and compared by the log-rank test. MTT method, plate colony formation assay, cell cycle, apoptosis, cell migration and invasion assays were tested using one-way ANOVA for factorial design. The significance of various variables for survival was analyzed by the Cox proportional-hazards model in the multivariate analysis. For real-time RT-PCR data analysis, the Student’s *t*-test was used to compare the values between subgroups in all cases. The SPSS 13.0 software was used for all statistical analyses and *p* < 0.05 was considered significant.

## SUPPLEMENTARY MATERIALS AND METHODS


