# Supplementary material for: The positive feedback between Snail and DAB2IP regulates EMT, invasion and metastasis in colorectal cancer
Source: Oncotarget. 2015 Aug 21;6(29):27427–39. doi: 10.18632/oncotarget.4861 (PMC4695000; doi:10.18632/oncotarget.4861)
Supplement: Supplementary file 1 [file oncotarget-06-27427-s001.pdf]

SUPPLEMENTARY MATERIALS AND METHODS

Cell lines and animals

Seven CRC cell lines with different metastatic abilities, including SW620, SW480, Lovo, LS174T, HT29, HCT116 and Caco-2 were obtained from the American Type. All cell lines were cultured in RPMI 1640 (Invitrogen) supplemented with 10% fetal calf serum (HyClone) and 1% penicillin/streptomycin (Invitrogen) in a 5% CO_2 humidified atmosphere at 37°C. For inhibitors treatment, 40 mM/L GSK-3β inhibitor LiCL (Sigma) or 5 μM/L proteasome inhibitor MG132 (CST) was added in the cultured cells for 24 hours. Four-to-six-week-old male athymic BALB/c-nu/nu mice were purchased from the Central Laboratory of Animal Science at Southern University (Guangzhou, China). The mice were maintained at our laboratory in a specific Pathogen Free environment. All protocols for animal studies were reviewed and approved by the Institutional Animal Care and Use Committee at our University.

Patients

This study was conducted on a total of 200 paraffin-embedded CRC samples from 2004 to 2005 year, which were histologically and clinically diagnosed from the Department of Pathology, Nanfang Hospital affiliated with Southern Medical University. They were not pretreated with radiotherapy or chemotherapy prior to surgery. They were followed up for 5 years and their complete clinical data were collected. For the use of these clinical materials for research purposes, prior patient's consent and approval from the Institute Research Ethics Committee were obtained. Clinical information of the samples was described in detail in Table 1. Patients included 111 males and 89 females, of ages ranging from 31 to 88 years (mean, 62 years old). All cases were with no metastasis at its presence at original presentation with CRC. The tables on metastasis pertain to its presence at any time in follow-up. A total of 75 (37.5%) patients died during follow up and 46 (23%) patients experienced distant metastasis. Fresh CRC tissues and the corresponding normal tissues were collected from 20 patients who underwent CRC resection without prior radiotherapy and chemotherapy at the Department of General Surgery in Nanfang Hospital in 2008 year. These samples were collected immediately after resection, snap-frozen in liquid nitrogen, and then stored at −80°C until needed.

Real-Time RT-PCR

Total RNA was extracted using Trizol reagent (Invitrogen) and cDNA was synthesized using an access RT system (Promega). Real-time PCR was performed using Mx3000P Real-time PCR System (Stratagene) and SYBR PremixExTaqTM (TaKaRa). The primers were selected as the following: For DAB2IP, forward 5′-TGG ACG ATG TGC TCT ATG CC-3′, reverse 5′-GGA TGG TGA TGG TTT GGT AG-3′. The PCR condition was 95°C for 30s, followed by 40 cycles of amplification (95°C for 5s, 60°C for 34s, 72°C for 34s). The comparative quantification was determined using the $2^{-\Delta\Delta Ct}$ method. Each sample was tested three times.

Western blot analysis

Proteins were extracted with RIPA buffer (1× PBS, 1%NP40, 0.1% SDS, 5 mM EDTA, 0.5% sodium deoxycholate, and 1 mM sodium orthovanadate) with protease inhibitors and quantified by BCA method. 50 μg proteins lysates were resolved on 10% SDS-PAGE, electrotransferred to polyvinylidene fluoride membranes (Immobilon P; Millipore, Bedford, MA). Membranes were immunoblotted overnight at 4°C with anti-DAB2IP polyclonal antibody (1:400, Abcam, Cambridge, UK), anti-β-Trcp, anti-H3 (Abcam, Cambridge, UK), anti-H3K27me3 (Abcam, Cambridge, UK), anti-snail, anti-EZH2, anti-HDAC1, anti-HDAC2, anti-GSK3β, anti-p-GSK3β (Cell Signaling Technology, Inc, USA), anti-E-Cadherin, anti-Vimentin, anti-β-catenin (Epitomics, USA), followed by their respective horseradish peroxidase-conjugated secondary antibodies. Signals were detected by enhanced chemiluminescence (Pierce, Rockford, IL).

Immunohistochemical Analysis (IHC)

The sections were deparaffinized and rehydrated, and endogenous peroxidase was inhibited with 0.3% H_2O_2 methanol. For antigen retrieval, slides were boiled in 0.01 M, pH 6.0 sodium citrate buffer for 15 min in a microwave oven. After blocked with the 5% normal goat serum, primary anti-DAB2IP polyclonal antibody (1:400, Abcam, Cambridge, UK), anti-Ezh2 monoclonal antibody (1:200, Cell Signal Technology, USA) and anti-Snail monoclonal antibody (Abcam, Cambridge, UK) was applied and the slides were incubated at 4°C overnight. Following incubation with biotinylated secondary antisera, the streptavidin-biotin complex/horseradish peroxidase was applied. Finally, the visualization signal was developed with DAB staining, and the slides were counterstained in hematoxylin. The stained tissue sections were reviewed and scored separately by two pathologists blinded to the clinical parameters. The staining intensity was scored as 0 (negative), 1 (weak), 2 (medium) and 3 (strong). The extent of staining was scored as 0 (0%),

1 (1–25%), 2 (26–50%), 3 (51–75%) and 4 (76–100%), according to the percentages of the positive staining areas in relation to the entire carcinoma-involved area or the entire section for the normal samples. The sum of the intensity and extent scores was used as the final staining score (0–7) for DAB2IP or Ezh2. The staining of DAB2IP or Ezh2 was assessed as follows: (−) means a final staining score of <3; (+) a final staining score of 3; (+ +) a final staining score of 4; and (+ + +) a final staining score of ≥5. Tumors having a final staining score of 3 or higher were considered to be positive. This relatively simple, reproducible scoring method gives highly concordant results between independent evaluators and has been used in previous studies [1, 2]. Cutoff values for DAB2IP were chosen based on a measure of heterogeneity by using log-rank statistical analysis with respect to overall survival. An optimal cutoff value was identified: Tumors with a final staining score 0~+ were classified as tumors with low expression of DAB2IP, and tumors with a final staining score ++~+++ were classified as tumors with high expression of DAB2IP.

Chromatin immunoprecipitation assay (ChIP)

Cells were lysed using SDS lysis buffer and DNA was sheared by sonication to lengths between 200 and 1000 base pairs. Protein-DNA complexes were precipitated by anti-snail antibody (Abcam, Cambridge, UK), and then recovered using protein G agarose beads, washed, and eluted. Crosslinks in protein-DNA complexes were then reversed by NaCL. The immunoprecipitated DNA was amplified by PCR for specific sequences containing snail-binding sites.

REFERENCES

1. Wang F, Qiao Y, Yu J, Ren X, Wang J, Ding Y, Zhang X, Ma W, Ding Y, Liang L. FBX Acts as an Invasion and Metastasis Suppressor and Correlates with Poor Survival in Hepatocellular Carcinoma. PloS one. 2013; 8:e65495.
2. Soumaoro LT, Uetake H, Higuchi T, Takagi Y, Enomoto M, Sugihara K. Cyclooxygenase-2 expression: a significant prognostic indicator for patients with colorectal cancer. Clinical cancer research : an official journal of the American Association for Cancer Research. 2004; 10:8465–8471.

SUPPLEMENTARY FIGURES AND TABLES

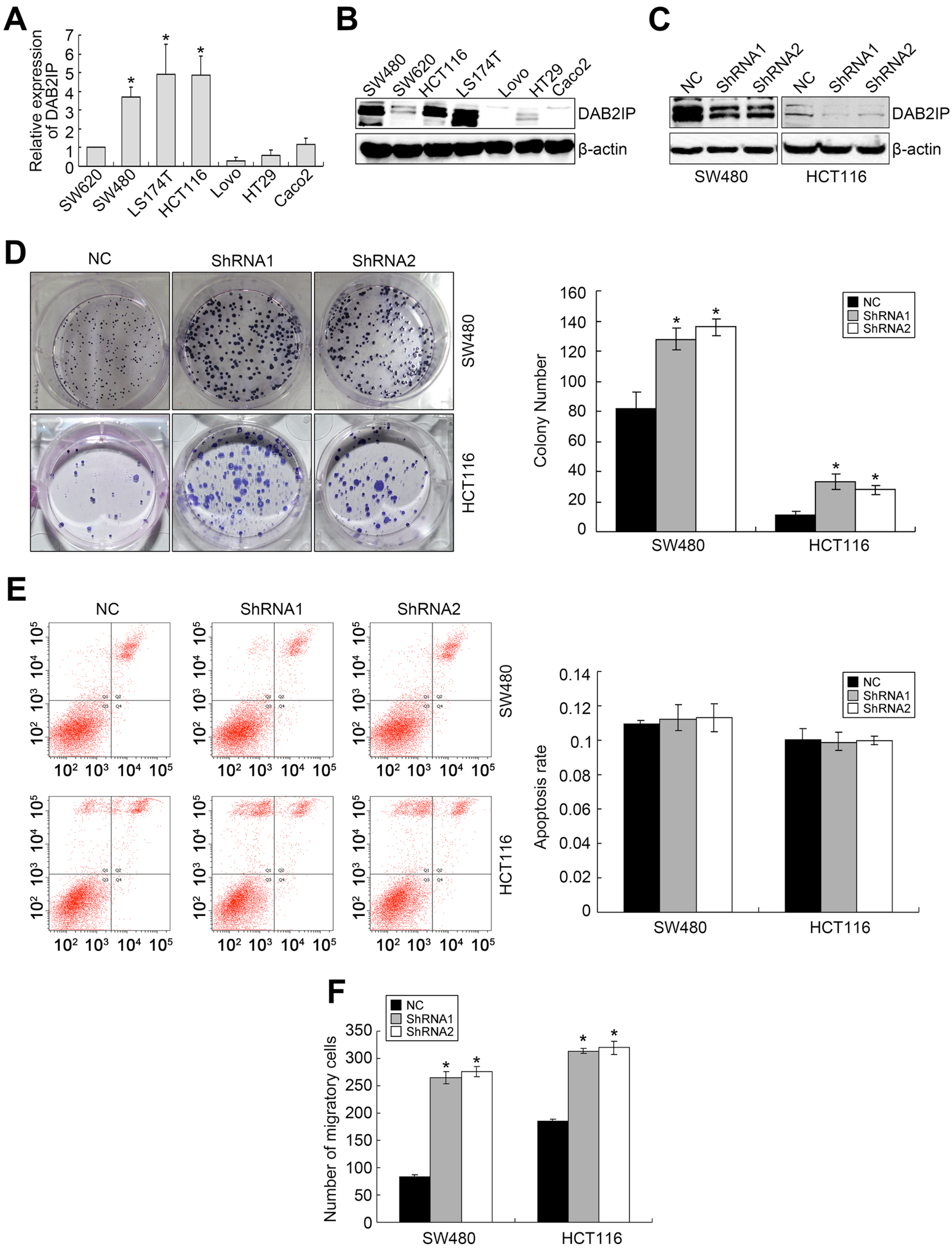

Supplementary Figure S1: DAB2IP is sufficient to inhibit proliferation, migration of CRC cells. A. Real-time RT-PCR analysis of endogenous DAB2IP expression in seven CRC cell lines. **B.** Western blot analysis of endogenous expression of DAB2IP in seven CRC cell lines. **C.** DAB2IP expression in SW480 and HCT116 cell lines transfected with two specific shRNAs toward DAB2IP by Western blotting. **D.** Effect of DAB2IP knockdown on the proliferation of SW480 and HCT116 cells by colony formation assay. **E.** Effect of DAB2IP knockdown on cell apoptosis of SW480 and HCT116 cells by Flow Cytometry. **F.** Effect of knockdown DAB2IP on the migration of SW480 and HCT116 cells by Transwell chamber.

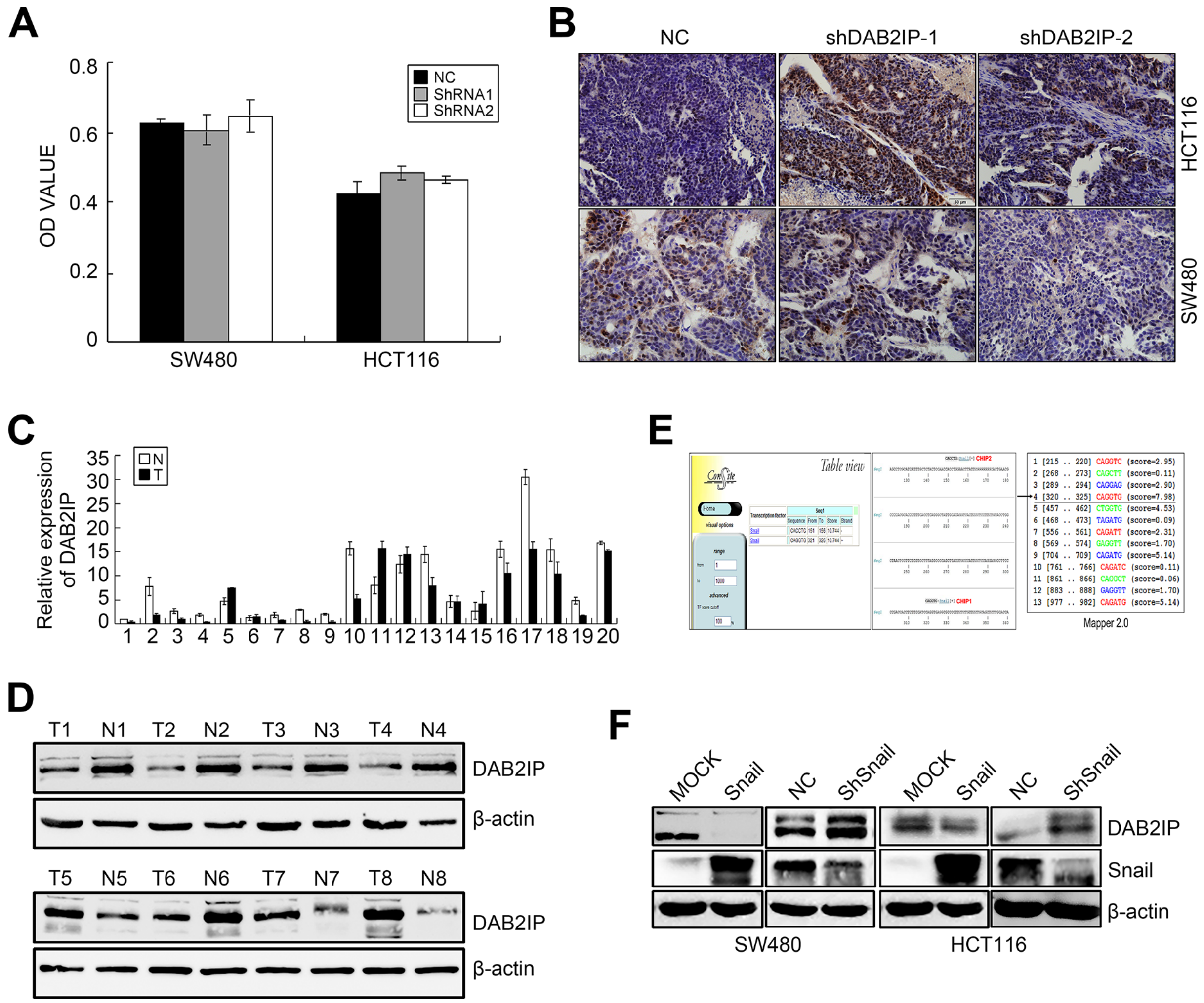

Supplementary Figure S2: DAB2IP is down-regulated in CRC tissues and EZH2 negatively regulates DAB2IP expression. A. Effect of knockdown DAB2IP on the adhesion of SW480 and HCT116 cells. $^*P < 0.05$. **B.** IHC staining of Ki-67 expression in subcutaneous tumors of mice injected with DAB2IP depleting SW480 and HCT116 cells. **C.** Real-time PCR analysis of DAB2IP expression in 20 paired CRC tissues. **D.** Western blot analysis of DAB2IP expression in 8 paired CRC tissues. **E.** Two possible binding motifs for snail within the promoter of DAB2IP were predicted by Consite, Mapper2.0, TRED databases, respectively. **F.** Expressions of DAB2IP and Snail expression in Snail-expressing or Snail-KD cells by Western blotting.

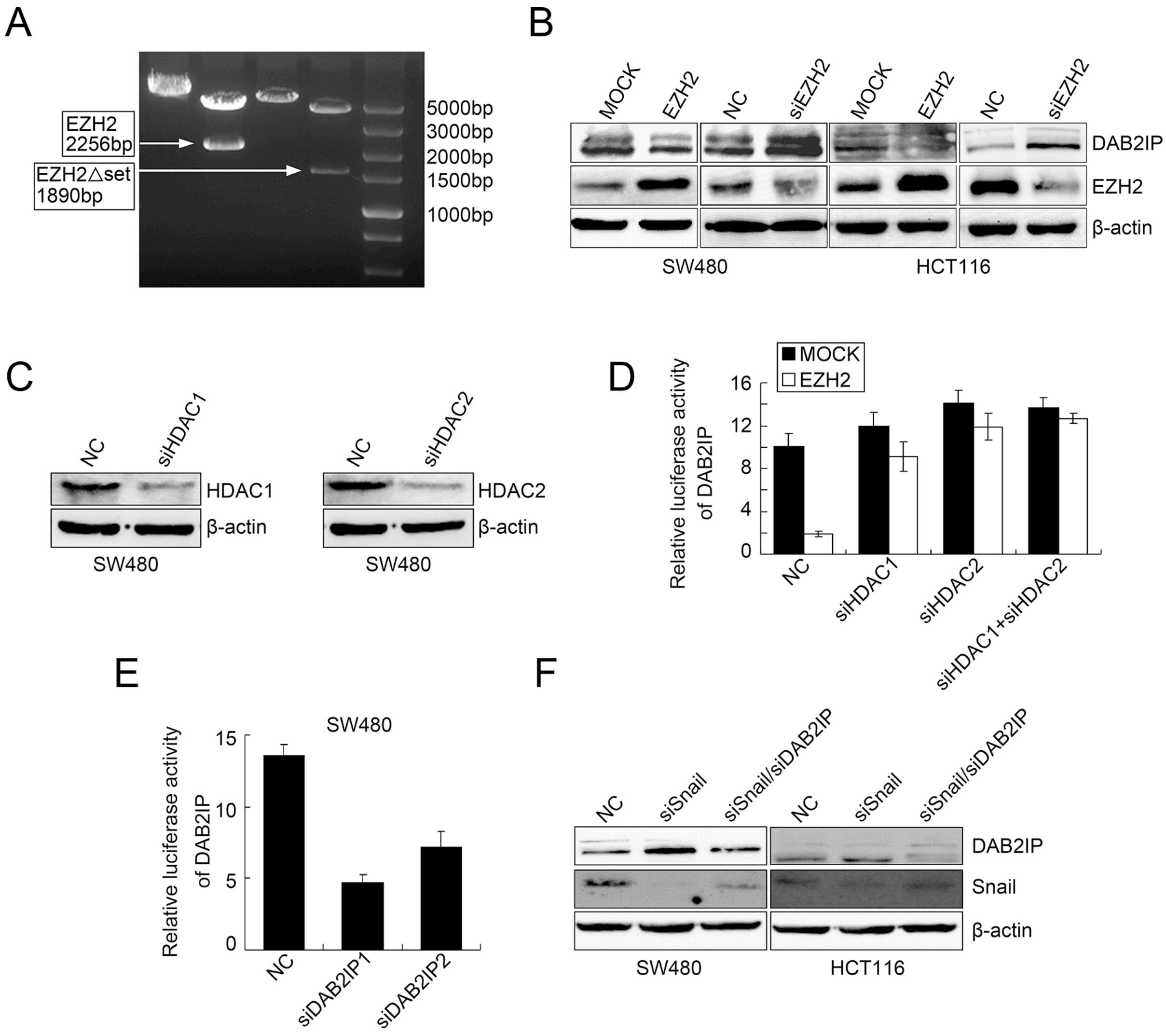

Supplementary Figure S3: EZH2 regulates DAB2IP expression dependent on HDAC1/2 activity and DAB2IP is required for Snail-induced the proliferation of SW480 and HCT116 cells. A. Constructions of EZH2 vector and the SET domain deletion mutation of EZH2 (EZH2ΔSET) vector. **B.** Effect of EZH2 over-expression and knockdown on the expression of DAB2IP by Western blotting. **C.** HDAC1/2 expressions in SW480 and HCT116 cell lines transfected with specific shRNAs toward HDAC1/2 by Western blotting. **D.** DAB2IP luciferase activity after transfection of siHDAC1, siHADC2 and siHDAC1+siHADC2 in SW480 cells. **E.** DAB2IP luciferase activity after transfection of DAB2IP siRNAs in SW480 cells. **F.** Snail and DAB2IP expressions in Snail- or Snail/DAB2IP-depleting SW480 and HCT116 cells by Western blotting.

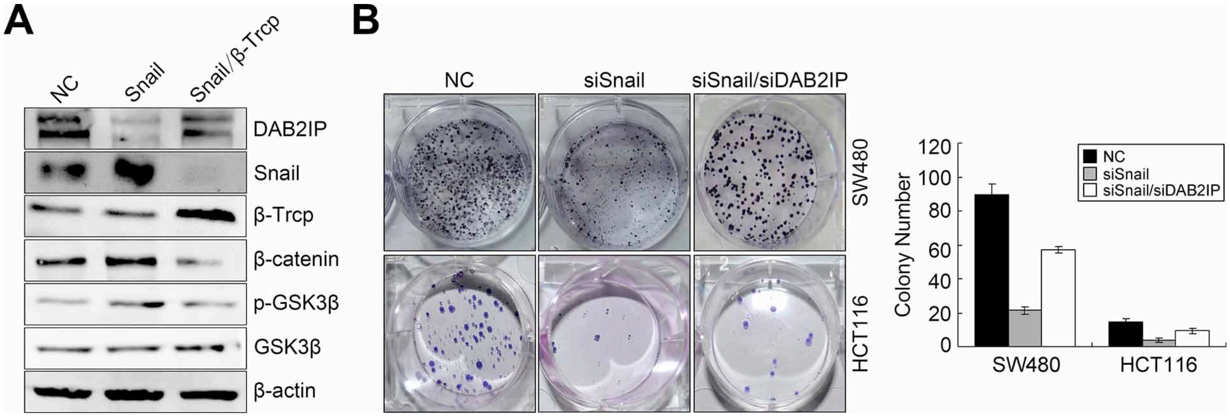

Supplementary Figure S4: EZH2 regulates DAB2IP expression dependent on HDAC1/2 activity and DAB2IP is required for Snail-induced the proliferation of SW480 and HCT116 cells. A. Expressions of DAB2IP, Snail, β-Trcp, β-catenin, p-GSK3β and GSK3β in Snail and Snail/β-Trcp overexpressing cells. **B.** Effects of Snail and Snail/DAB2IP knockdown on the proliferation of SW480 and HCT116 cells by colony formation assay.

Supplementary Table S1: Relationship between DAB2IP expressions and clinicopathologic features of CRC patients

Features	Total Number	High expression	Low expression	*P*	χ^2
All case	200	66	134		
Age				0.980	0.010
<55	124	41	83		
>=55	76	25	51		
Gender				0.473	0.514
Male	111	39	72		
Female	89	27	62		
Tumor size				0.851	0.035
<5 cm	86	29	57		
>=5 cm	114	37	77		
Differentiation				***0.007***	9.892
Well	62	17	45		
Moderate	91	40	51		
Poor	47	9	38		
Serosal Invasion				0.249	1.331
N	26	6	20		
Y	174	60	114		
Lymphatic Metastasis				***0.000***	14.965
N	126	54	72		
Y	74	12	62		
LNR				0.244	1.355
N	181	62	119		
Y	19	4	15		
Dukes' stage				***0.000***	13.753
A + B	99	45	54		
C + D	101	21	80		
Remote Metastasis				***0.010***	6.583
N	154	58	96		
Y	46	8	38		

Abbreviation: LNR, lymph node ratio

Supplementary Table S2: Univariate and multivariate analyses of individual parameters for correlations with overall survival rate: Cox proportional hazards model

Variables	Univariate		*P* value	Multivariate		*P* value
	HR	CI (95%)		HR	CI (95%)	
DAB2IP	0.323	0.174–0.599	0.000*	0.396	0.212–0.741	0.004*
Age	0.965	0.604–1.541	0.880			
Gender	0.748	0.474–1.180	0.212			
Tumor Size	0.757	0.475–1.207	0.243	0.643	0.401–1.032	0.067
Tumor Grade	0.606	0.439–0.838	0.002*			
Serosal Invasion	0.180	0.588–2.371	0.641			
Dukes' stage	2.898	1.761–4.770	0.000*	2.658	1.594–4.434	0.000*

Abbreviations: HR, Hazard radio; CI, Confidence interval.
[1]Statistically significant ($p < 0.05$).
